# Impact of Concomitant Impairments of the Left and Right Ventricular Myocardial Strain on the Prognoses of Patients With ST-Elevation Myocardial Infarction

**DOI:** 10.3389/fcvm.2021.659364

**Published:** 2021-05-31

**Authors:** Wei Lai, He Jie, Dong Jian-Xun, Kong Ling-Cong, Zeng Jun-Tong, Shi Bo-Zhong, An Dong-Ao-Lei, Chen Bing-Hua, Ding Song, Li Zheng, Yang Fan, Yang Yi-Ning, Yan Fu-Hua, Xiu Jian-Cheng, Wang Hu-Wen, Xu Jian-Rong, Ge Heng, Pu Jun

**Affiliations:** ^1^Department of Cardiology, School of Medicine, Renji Hospital, Shanghai Jiao Tong University, Shanghai, China; ^2^Department of Radiology, School of Medicine, Renji Hospital, Shanghai Jiao Tong University, Shanghai, China; ^3^The First Affiliated Hospital, Xinjiang Medical University, Ürümqi, China; ^4^Department of Radiology, School of Medicine, Ruijin Hospital, Shanghai Jiao Tong University, Shanghai, China; ^5^Nanfang Hospital, Southern Medical University, Guangzhou, China; ^6^School of Public Health, Shanghai Jiaotong University, Shanghai, China

**Keywords:** ST-elevation myocardial infarction, cardiac magnetic resonance, myocardial strain analysis, right ventricle, prognostic implications

## Abstract

**Background:** The impact of concomitant impairments of left and right ventricular (LV and RV) strain on the long-term prognosis of acute ST-elevation myocardial infarction (STEMI) is not clear.

**Methods:** We analyzed CMR images and followed up 420 first STEMI patients from the EARLY Assessment of MYOcardial Tissue Characteristics by CMR in STEMI (EARLY-MYO-CMR) registry (NCT03768453). These patients received timely primary percutaneous coronary intervention (PCI) within 12 h and CMR examination within 1 week (median, 5 days; range, 2–7 days) after infarction. Global longitudinal strain (GLS), global radial strain (GRS), and global circumferential strain (GCS) of both ventricles were measured based on CMR cine images. Conventional CMR indexes were also assessed. Primary clinical outcome was composite major adverse cardiac and cerebrovascular events (MACCEs) including cardiovascular death, re-infarction, re-hospitalization for heart failure and stroke. In addition, CMR data from 40 people without apparent heart disease were used as control group.

**Results:** Compared to controls, both LV and RV strains were remarkably reduced in STEMI patients. During follow-up (median: 52 months, interquartile range: 29–68 months), 80 patients experienced major adverse cardiac and cerebrovascular events (MACCEs) including cardiovascular death, re-infarction, heart failure, and stroke. LV-GCS > −11.20% was an independent predictor of MACCEs (*P* < 0.001). RV-GRS was the only RV strain index that could effectively predict the risk of MACCEs (AUC = 0.604, 95% CI [0.533, 0.674], *P* = 0.004). Patient with RV-GRS ≤ 38.79% experienced more MACCEs than those with preserved RV-GRS (log rank *P* < 0.001). Moreover, patients with the concomitant decrease of LV-GCS and RV-GRS were more likely to experience MACCEs than patients with decreased LV-GCS alone (log rank *P* = 0.010). RV-GRS was incremental to LV-GCS for the predictive power of MACCEs (continuous NRI: 0.327; 95% CI: 0.095–0.558; *P* = 0.006). Finally, tobacco use (*P* = 0.003), right coronary artery involvement (*P* = 0.002), and LV-GCS > −11.20% (*P* = 0.012) was correlated with lower RV-GRS.

**Conclusions:** The concomitant decrease of LV and RV strain is associated with a worse long-term prognosis than impaired LV strain alone. Combination assessment of both LV and RV strain indexes could improve risk stratification of patients with STEMI.

**Trial Registration:** ClinicalTrials.gov, NCT03768453. Registered 7 December 2018 - Retrospectively registered, https://clinicaltrials.gov/ct2/show/NCT03768453.

## Introduction

The in-hospital mortality rate of patients with acute ST-elevation myocardial infarction (STEMI) has greatly declined (1). However, the long-term prognosis of patients with STEMI is quite varied (2). Therefore, the early risk stratification of patients with STEMI is essential. Accurate and clinically applicable indicators are needed.

The left ventricle (LV) function is a key prognostic factor for patients with STEMI (2). Although LV ejection fraction (LVEF) is regularly used as a measurement of LV function, it is less sensitive for the detection of regional or subtle myocardial impairments, which are common in the early phase of myocardial infarction and occur as the very beginning of subsequent adverse cardiac remodeling (3). For the past decade, myocardial strain measurements have been used to determine LV function. Myocardial strain demonstrates the absolute magnitude of myocardial deformation in each segment compared to the global estimation of conventional LVEF (3). Recent studies have shown that the impairments of LV strains measured by echocardiography and cardiac magnetic resonance (CMR) in the acute phase of STEMI are closely related to future LV remodeling, sustained deterioration of LVEF, and the occurrence of adverse events (4–6).

The impairment of right ventricular (RV) function may also help determine the prognosis of patients with STEMI (7–9). However, the irregular anatomic morphology renders the visualization of the RV so that accurate measurement of its strains by echocardiography remains challenging. The inter- and intra-variability of the measurements found in echocardiographic studies likely attributes to the conflicting results (10). Therefore, the prognostic impact of concomitant RV dysfunction in STEMI patients remains undetermined.

In this study, we used feature-tracking (FT) technology based on the CMR cine images of patients with STEMI obtained within their first week of hospitalization and followed the prognosis of each patient. We aimed to investigate the prognostic implication of combinative assessment of both LV and RV strain indexes in patients with STEMI.

## Methods

### Study Design and Subjects

**Figure 1:**
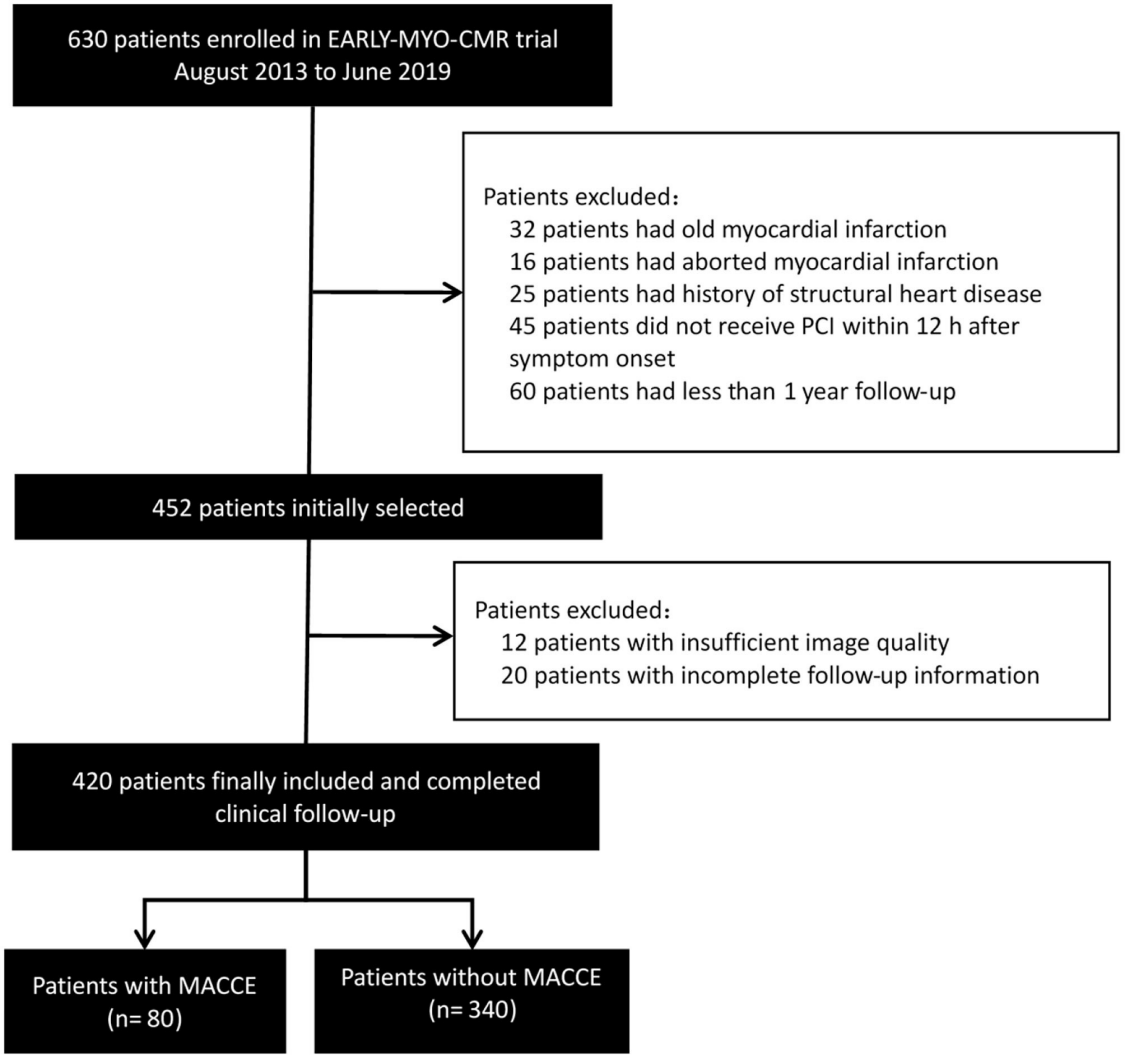
Flowchart of study inclusion. MACCE, major adverse cardiac and cerebrovascular events; PCI, percutaneous coronary intervention.

We used patient data obtained from the Early Assessment of Myocardial Tissue Characteristics by CMR in STEMI Registry (*EARLY-MYO-CMR*, NCT03768453). The multi-center registry prospectively includes all-comer patients with STEMI who have undergone CMR imaging (11). The inclusion criteria for the current study were as follows: first-time STEMI (as diagnosed by typical ischemic syndrome and electrocardiography (EKG) manifestation of ST elevation in at least two contiguous precordial (≥2 mm) or peripheral leads (≥1 mm), primary percutaneous coronary intervention (PCI) within 12 hours after symptom onset, and CMR imaging within the first week of STEMI onset. All patients received standard medical care (2). The flowchart of study inclusion is seen in Figure 1. In addition, CMR data from 40 people without apparent heart disease were used as control group.

### CMR Imaging and Analysis

CMR imaging was performed using 3.0-Tesla scanners (Achieva TX, Philips Healthcare, Netherlands). All sequences were acquired in breath-hold with a default field of view (FOV) at 350*350 mm^2^. A balanced, steady-state free precession (SSFP) sequence was used to produce cine images (TR/TE 3.2/1.6 ms, 30 phases, flip angle 45°, voxel size 2.0*1.6*8 mm^3^). A short-tau inversion-recovery spin echo sequence was used to produce T2-weighted black-blood images (TR/TE 2 R-R intervals/75 ms, voxel size 2.0*1.6*8 mm^3^). Ten minutes after the administration of 0.1 mmol/kg gadopentetate dimeglumine (Magnevist, Bayer HealthCare Pharmaceuticals Inc., Wuppertal, Germany), late gadolinium enhancement (LGE) images were acquired using an inversion recovery segmented SSFP sequence with a proper inversion time (TR/TE 3.5/1.7 ms, flip angle 25°, temporal resolution 190 ms, voxel size 1.5*1.7*10 mm^3^ interpolated into 0.74*0.74*5 mm^3^).

**Figure 2:**
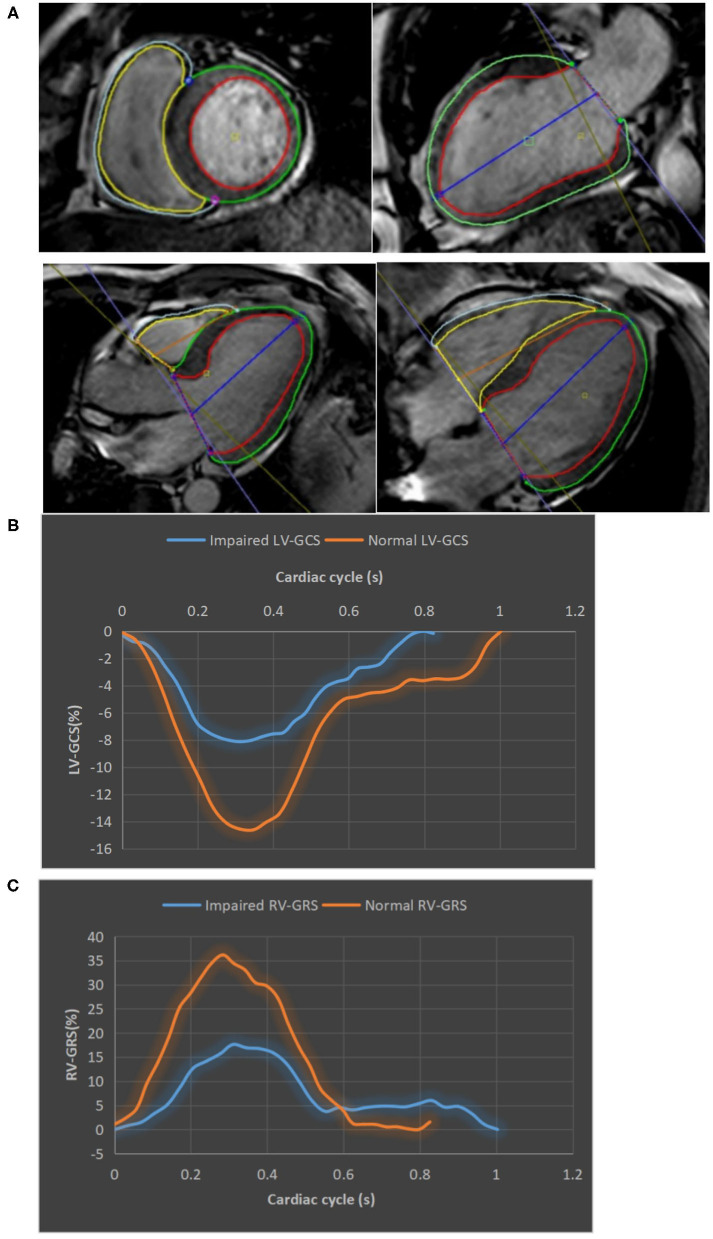
The measurement of LV/RV strains. **(A)** LV and RV tracking in cine CMR short axis, two-, three-, and four-chamber views. The epicardial and endocardial contour of LV/RV were delineated by green, red, gray, and yellow lines, respectively. **(B)** Strain curves of a patient with impaired LV-GCS (−8.13%) and a patient with normal LV-GCS (−14.62%). **(C)** Strain curves of a patient with impaired RV-GRS (17.61%) and a patient with normal RV-GRS (36.15%). CMR, cardiac magnetic resonance; GCS, global circumferential strain; GRS, global radial strain.

Images were analyzed offline by an experienced reader blinded to the patients' clinical data using commercial software (CVI42, Circle Cardiovascular Imaging, Inc., Calgary, Canada). Contours were acquired according to an established protocol in the core lab (11, 12). The software automatically delineated the borders of the epicardium, endocardium, infarction, intramyocardial hemorrhage (IMH), and microvascular obstructions (MVO). All contours were inspected and manual corrections were made when necessary. Volumetric parameters, such as LVEF, were calculated using short-axis cine images covering the whole heart. Infarction was determined as hyperenhanced myocardium (a signal intensity > 5 SDs of normal myocardium). The extent of the infarction and MVO were quantified as a percentage of left ventricular myocardial mass (%LV). FT analysis was performed based on the cine images according to previously reported protocols (5, 13). For LV strain, data were derived from contiguous short-axis images (slice thickness: 8 mm, gap: 0 mm) and single-slice long-axis views (2-chamber, 3-chamber, and 4-chamber planes). The software calculated segmental peak strains on a 16-segment model. Subsequently, the global strains, including global longitudinal strain (GLS), global radial strain (GRS), and global circumferential strain (GCS), were calculated as the mean of the respective segmental peak values. The RV-GLS, GRS, and GCS and the RVEF were measured using the short-axis, 3-chamber and 4-chamber long-axis images. Contours of the RV endocardium and epicardium were manually delineated. Figure 2 shows the measurement of LV/RV strain and typical strain changes in patients with normal or impaired LV/RV function. Besides, 10% of the cases were randomly selected to test the intra- and inter-observer variability of the CMR analysis.

### Angiographic Assessments

Angiographic assessments, including the thrombolysis in myocardial infarction (TIMI) myocardial flow grading (TFG) (14), were performed according to their standard protocols. An experienced interventional cardiologist analyzed all of the images.

### Clinical End Points and Follow-Up

The clinical end point of the study was the incidence of composite major adverse cardiac and cerebrovascular events (MACCEs). MACCEs were defined as cardiovascular death, non-fatal myocardial re-infarction, hospitalization for heart failure, and stroke after discharge (15). Clinical follow-up was performed through telephone or clinical interviews by two study nurses blinded to the research data at day 30 and months 3, 6, and 12 post-STEMI, and every 6 months thereafter. Reported MACCEs were confirmed using the patients' electronic medical records.

### Statistical Analysis

The distribution of data was determined using the Kolmogorov-Smirnov test. Normally distributed continuous variables are presented as mean ± SD and compared using the Student's *t*-test. Variables without normal distribution are presented as median and interquartile range (IQR) and compared using the Wilcoxon rank sum test. Categorical variables are presented as number of cases with corresponding percentages and the differences were compared using the Chi-square test or the Fisher's exact test. The correlation between two continuous variables was evaluated using the Spearman's coefficient. The intra- and inter-observer variability of CMR analysis was assessed using intra-class correlation coefficients (ICC) and Bland-Altman analysis. An ICC > 0.74 was considered high consistency.

The factors influencing the right ventricular strain were determined using logistic regressions. Kaplan-Meier curves were used to illustrate event-free rates, and the differences were compared using the log-rank test. The predictors of MACCEs were determined using multivariable Cox regression analysis. Statistically significant (*p* ≤ 0.05) variables in the univariate Cox regression model and other clinically significant variables were included in the multivariate analysis. The discriminative power of the predictors of MACCEs were further assessed using receiver operating characteristic analyses with the optimal cutoff points of continuous MACCEs predictors estimated by the Youden Index. All analyses in this study were performed using SPSS version 23.0 (SPSS Inc., Chicago, Illinois). The additional predictive power of the incidence of MACCEs for concomitantly impaired LV-GCS and RV-GRS compared with that of impaired LV-GCS alone was further assessed using the reclassification analyses (R package PredictABLE software, R version 3.6.2, the R Foundation, Vienna, Austria). The continuous net reclassification improvement (NRI), which indicates improvement of the prediction, was determined. Statistical significance was set at *P* < 0.05.

## Results

A total of 420 patients in the Early-MYO-CMR database met the inclusion criteria of this analysis (Figure 1). Among them, 310 patients had been included in a previous analysis (15). CMR imaging was performed a median of 5 days (range: 2–7 days) after symptom onset.

### Clinical Characteristics and MACCEs

During a median follow-up of 52 months (IQR: 29–68 months), 80 patients (19.0%) experienced MACCEs, including 8 cardiovascular deaths (1.9%), 19 myocardial re-infarctions (4.5%), 46 admissions for heart failure (10.9%), and 7 strokes (1.7%). Table 1 shows the patients' clinical characteristics. Patients who experienced MACCEs had a higher prevalence of hypertension, higher Killip classification on admission, and worse TFG after PCI. The peak concentrations of creatine kinase, the MB isoenzyme of CK, and high-sensitivity cardiac troponin I were significantly higher in patients who experienced MACCEs. Patients who experienced MACCEs received more stents than those who did not.

**Table 1:** Clinical characteristics of patients with STEMI.

**Parameter**	**All**	**Non-MACCE**	**MACCE**	***P*-value**
	**(*n* = 420)**	**(*n* = 340)**	**(*n* = 80)**	
Age, y	60 (54, 65)	60 (54, 65)	59 (54, 65)	0.754
Male, *n* (%)	375 (89.3)	306 (90.0)	69 (86.3)	0.329
BMI, kg/m^2^	24.96 (22.86, 26.67)	24.82 (22.76, 26.57)	25.10 (22.90, 27.68)	0.379
Total ischemic time, h	4.50 (3.23, 5.85)	4.50 (3.14, 5.71)	4.70 (3.75, 6.44)	0.185
Hypertension, *n* (%)	234 (55.7)	176 (51.8)	58 (72.5)	**0.001**
Diabetes, *n* (%)	129 (30.7)	99 (29.1)	30 (37.5)	0.144
Smoking, *n* (%)	245 (58.3)	192 (56.5)	53 (66.3)	0.110
Hyperlipidemia, *n* (%)	261 (62.1)	212 (62.4)	49 (61.3)	0.855
Systolic blood pressure on admission, mmHg	136 (122, 150)	136 (122, 150)	137 (123, 150)	0.991
Heart rate on admission, bpm	78 (68, 88)	77 (67, 87)	80 (72, 88)	0.059
Killip classification on admission, *n* (%)				**0.016**
1	391 (93.0)	324 (95.3)	67 (83.8)	
2	19 (4.6)	13 (3.8)	6 (7.5)	
3	6 (1.4)	2 (0.6)	4 (5.0)	
4	4 (1.0)	1 (0.3)	3 (3.7)	
TIMI flow pre-PCI, *n* (%)				0.436
0–1	242 (57.6)	199 (58.5)	43 (53.8)	
2–3	178 (42.4)	141 (41.5)	37 (46.3)	
TIMI flow post-PCI, *n* (%)				**0.007**
0–2	40 (9.5)	26 (7.6)	14 (17.5)	
3	380 (90.5)	314 (92.4)	66 (82.5)	
Culprit vessel, *n* (%)				0.410
LAD	247 (58.8)	195 (57.4)	52 (65.0)	
LCX	37 (8.8)	32 (9.4)	5 (6.3)	
RCA	136 (32.4)	113 (33.2)	23 (28.8)	
Number of affected vessels, *n* (%)				0.651
1	188 (44.8)	154 (45.3)	34 (42.5)	
≥2	232 (55.2)	186 (54.7)	46 (57.5)	
Number of stents, *n* (%)				**0.045**
0–1	315 (75.0)	262 (77.1)	53 (66.3)	
≥2	105 (25.0)	78 (22.9)	27 (33.8)	
Peak hs-CRP, mg/L	10.40 (3.30, 25.70)	9.46 (3.32, 23.35)	13.70 (3.05, 36.10)	0.064
Peak CK, U/L	2,223 (1,029, 3,792)	1,967 (896, 3,471)	3,429 (1,853, 5,151)	**<0.001**
Peak CK-MB, U/L	229.24 (94.08, 372.88)	198.15 (81.63, 339.43)	376.10 (235.13, 568.28)	**0.025**
Peak hs-cTnI, ng/ml	26.57 (6.76, 55.79)	23.80 (6.86, 49.93)	36.29 (5.02, 99.21)	**<0.001**
Peak D-dimer, ug/ml	0.13 (0.09, 0.24)	0.13 (0.09, 0.24)	0.16 (0.11, 0.27)	0.060
HbAc, %	5.8 (5.4, 6.7)	5.8 (5.4, 6.6)	5.8 (5.4, 7.0)	0.427
LDL-C, mmol/L	3.15 (2.57, 3.78)	3.13 (2.54, 3.78)	3.20 (2.72, 3.74)	0.469
Scr, umol/L	74.00 (65.13, 85.85)	74.00 (66.00, 85.30)	71.95 (61.13, 86.75)	0.386
ALT, U/L	35.00 (22.00, 54.00)	34.10 (22.00, 51.53)	38.55 (25.25, 66.43)	0.067

*ALT, alanine transaminase; BMI, body mass index; BNP, B-type natriuretic peptide; CK, creatine kinase; CK-MB, MB isoenzyme of creatine kinase; HbAc, glycosylated hemoglobin; hs-CRP, high-sensitivity C-reactive protein; hs-cTnI, high-sensitivity cardiac troponin I; LAD, left anterior descending artery; LCX, left circumflex artery; LDL-C, low density lipoprotein cholesterol; MACCE, major adverse cardiac and cerebrovascular events; PCI, percutaneous coronary intervention; RCA, right coronary artery; Scr, serum creatinine; STEMI, ST-elevation myocardial infarction; TIMI, thrombolysis in myocardial infarction. P-values of factors with bold values are less than 0.05*.

### CMR Indexes and MACCEs

Compared to the control group, participants with STEMI had significantly lower LV and RV strains (LVGLS:−8.39% vs. −13.07%, *P* < 0.001; LVGRS: 18.66% vs. 33.65%, *P* < 0.001; LVGCS:−14.20% vs. −20.81%, *P* < 0.001; RVGLS: −11.49% vs. −22.54%, *P* = 0.010; RVGRS: 41.84% vs. 54.38%, *P* < 0.001; RVGCS: −13.09% vs. −18.93%, *P* = 0.017).

**Figure 3:**
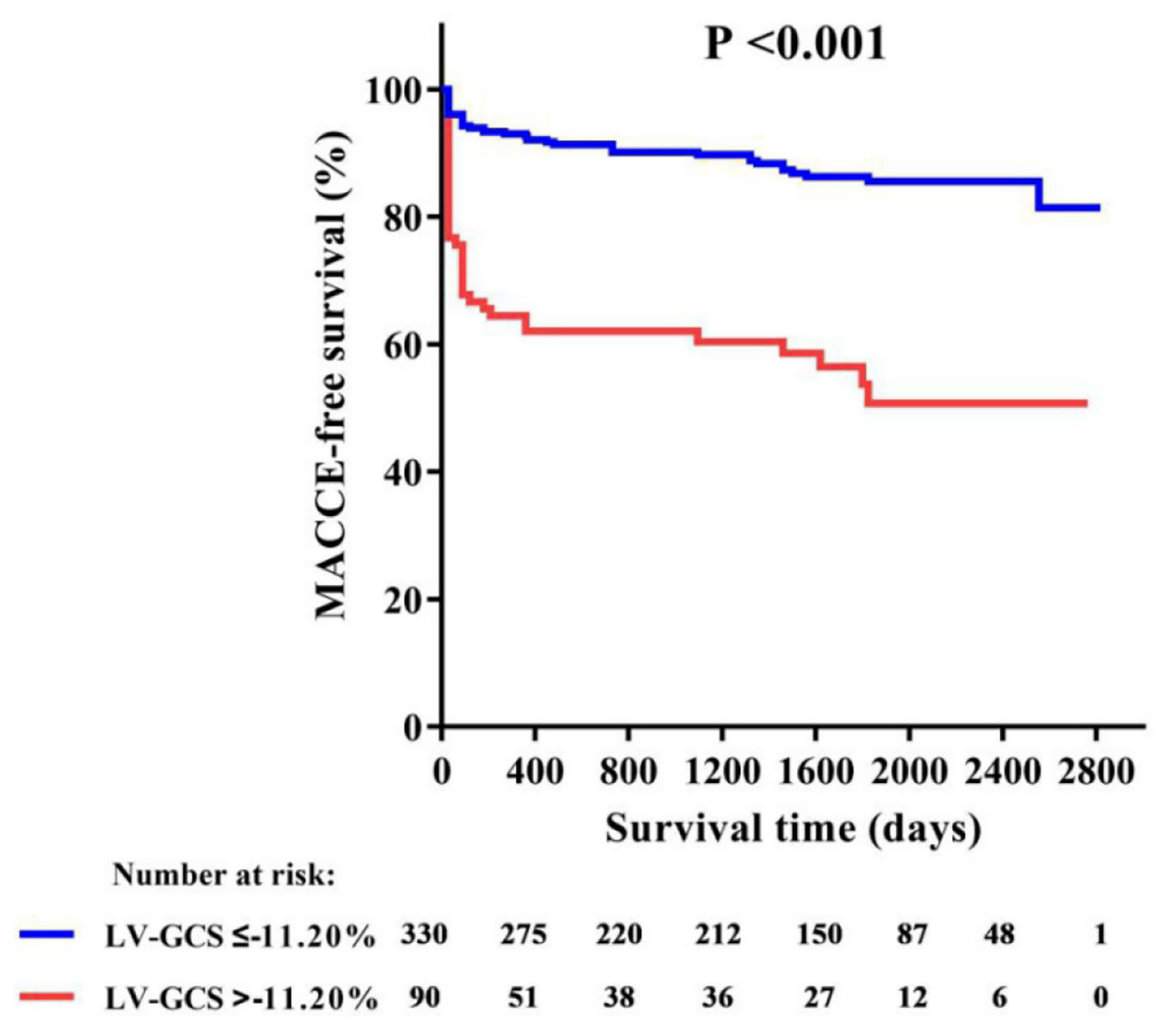
Kaplan-Meier curve of MACCE-free survival in patients with or without LV-GCS impairment. GCS, global circumferential strain; MACCE, major adverse cardiac and cerebrovascular events.

Table 2 shows the patient's CMR indexes. In general, the patients who developed MACCEs presented with a higher degree of LV enlargement, lower LVEF, more extensive infarctions and MVO, and a higher incidence of aneurysms and thrombus formation in the LV. All 3 LV strain indexes and RV-GRS were significantly decreased in those patients. LV-GCS was found to be an independent predictive factor of MACCEs in the multivariate Cox regression models adjusting for clinical characteristics and CMR indexes (Table 3). The ROC analysis determined that the optimal cut-off point of LV-GCS to predict MACCEs was −11.20% (Youden's index: 0.703; 95% CI: 0.634–0.772; *P* < 0.001), with a sensitivity of 48.75% and a specificity of 85.00%. Patients with LV-GCS > −11.20% were significantly more likely to experience MACCEs than patients with LV-GCS ≤ −11.20% (43.3% vs. 12.4%, *P* < 0.001). The predictive power of LV-GCS for MACCEs at this cutoff was more accurate than that of LVEF (area under the curve: 0.703 and 95% CI: 0.634–0.772 vs. 0.630 and 95% CI: 0.557–0.703, *P* = 0.003). The Kaplan-Meier curves for MACCEs-free survival were significantly different between patients with LV-GCS beyond or below the cutoff (*P* < 0.001 by log-rank test) (Figure 3).

**Table 2:** CMR indexes of patients with STEMI.

**Parameter**	**All (*n* = 420)**	**Non-MACCE (*n* = 340)**	**MACCE (*n* = 80)**	***P*-value**
LV-GRS, %	18.66 (13.85, 24.55)	19.57 (15.18, 25.15)	14.38 (10.10, 19.94)	**<0.001**
LV-GCS, %	−14.20 (−17.08, −11.51)	−14.72 (−17.25, −12.23)	−11.60 (−14.20, −8.70)	**<0.001**
LV-GLS, %	−8.39 (−10.70, −6.52)	−8.61 (−10.85, −6.63)	−7.68 (−9.33, −6.15)	**0.018**
RV-GRS, %	41.84 (33.10, 54.29)	43.88 (34.05, 55.74)	37.23 (29.95, 47.17)	**0.004**
RV-GCS, %	−13.09 (−15.74, −10.49)	−13.17 (−15.76, −10.27)	−12.57 (−15.72, −10.79)	0.801
RV-GLS, %	−11.49 (−14.14, −8.32)	−11.72 (−14.21, −8.47)	−10.57 (−13.91, −7.90)	0.239
LV-EDV, ml	115.64 (99.72, 134.56)	114.78 (98.62, 131.96)	121.69 (101.10, 146.46)	**0.028**
LV-ESV, ml	46.85 (36.12, 61.75)	45.20 (35.82, 58.08)	57.77 (38.48, 83.92)	**<0.001**
LV-SV, ml	66.71 (55.23, 76.94)	67.63 (55.81, 77.45)	64.09 (51.63, 75.40)	0.095
LV-EF, %	58.48 (50.26, 65.45)	59.63 (52.01, 65.79)	52.94 (41.82, 63.92)	**<0.001**
RV-EDV, ml	84.60 (68.74, 104.64)	84.62 (68.96, 105.07)	84.14 (66.85, 102.35)	0.618
RV-ESV, ml	35.46 (26.21, 45.17)	35.53 (26.65, 45.40)	34.79 (23.41, 42.67)	0.485
RV-SV, ml	48.78 (36.18, 63.12)	48.78 (36.36, 63.66)	48.53 (33.95, 59.86)	0.483
RV-EF, %	58.26 (50.10, 65.30)	58.35 (50.14, 64.98)	58.07 (49.79, 68.49)	0.918
LV-IS, % of LVMM	24.50 (17.31, 33.32)	23.52 (16.71, 31.00)	30.68 (20.97, 41.75)	**<0.001**
LV-MVO, % of LVMM	1.10 (0, 2.90)	0.90 (0, 2.60)	2.04 (0.15, 5.54)	**0.001**
LV IMH, *n* (%)	236 (56.2)	185 (54.4)	51 (63.8)	0.130
LV thrombus, *n* (%)	34 (8.1)	22 (6.5)	12 (15.0)	**0.012**
LV aneurysm, *n* (%)	114 (27.1)	82 (24.1)	32 (40.0)	**0.004**

*EDV, end-diastolic volume; EF, ejection fraction; ESV, end-systolic volume; GCS, global circumferential strain; GLS, global longitudinal strain; GRS, global radial strain; IMH, Intramyocardial hemorrhage; IS, infarct size; LVMM, left ventricular myocardial mass; MVO, microvascular obstruction; SV, stroke volume; MACCE, major adverse cardiac and cerebrovascular events. P-values of factors with bold values are less than 0.05*.

**Table 3:** Cox regression analysis of predictors for MACCE.

**Parameter**	**Univariate analysis**		**Multivariate analysis**	
	**HR (95% CI)**	***P***	**HR (95% CI)**	***P***
LV-GCS, %	1.212 (1.140, 1.287)	**<0.001**	1.157 (1.082, 1.237)	**<0.001**
LV-GRS, %	0.919 (0.887, 0.952)	**<0.001**		
LV-GLS, %	1.085 (1.013, 1.162)	**0.020**		
RV-GCS, %	1.013 (0.957, 1.071)	0.661		
RV-GRS, %	0.989 (0.975, 1.002)	0.105		
RV-GLS, %	1.042 (0.986, 1.101)	0.143		
Male, *n* (%)	0.695 (0.368, 1.315)	0.264		
Age	1.000 (0.976, 1.025)	0.984		
Hypertension, *n* (%)	2.301 (1.408, 3.760)	**0.001**	1.913 (1.105, 3.311)	**0.020**
Diabetes, *n* (%)	1.414 (0.899, 2.224)	0.134		
Smoking, *n* (%)	1.254 (0.787, 1.997)	0.342		
Heart rate on admission, bpm	1.016 (1.001, 1.032)	**0.039**		
Killip classification on admission, *n* (%)				
1	Ref	–		
2	1.893 (1.166, 3.075)	**0.010**		
3	2.621 (1.232, 5.575)	**0.012**		
4	2.932 (0.709, 12.122)	0.137		
TIMI flow post-PCI, *n* (%)				
0–2	1.989 (1.117, 3.543)	**0.020**		
3	Ref	–		
Culprit vessel, *n* (%)				
LAD	Ref	–		
LCX	0.579 (0.231, 1.449)	0.243		
RCA	0.761 (0.466, 1.243)	0.275		
Peak hs-cTnI, ng/ml	1.011 (1.005, 1.017)	**<0.001**		
LV-EDV, ml	1.008 (1.001, 1.014)	**0.018**		
LV-EF, %	0.960 (0.945, 0.976)	**<0.001**		
LV-IS, % LVMM	1.045 (1.027, 1.062)	**<0.001**		
LV-MVO, % LVMM	1.153 (1.096, 1.213)	**<0.001**	1.079 (1.016, 1.147)	**0.013**
LV thrombus, *n* (%)	2.301 (1.244, 4.253)	**0.008**		
LV aneurysm, *n* (%)	2.066 (1.319, 3.235)	**0.002**		

*EDV, end-diastolic volume; EF, ejection fraction; GCS, global circumferential strain; GLS, global longitudinal strain; GRS, global radial strain; hs-cTnI, high-sensitivity cardiac troponin I; LVMM, left ventricular myocardial mass; MACCE, major adverse cardiac and cerebrovascular events; MVO, microvascular obstruction; PCI, percutaneous coronary intervention; TIMI, thrombolysis in myocardial infarction; IS, infarct size. Factors with bold values are included in the multivariate analysis*.

**Figure 4:**
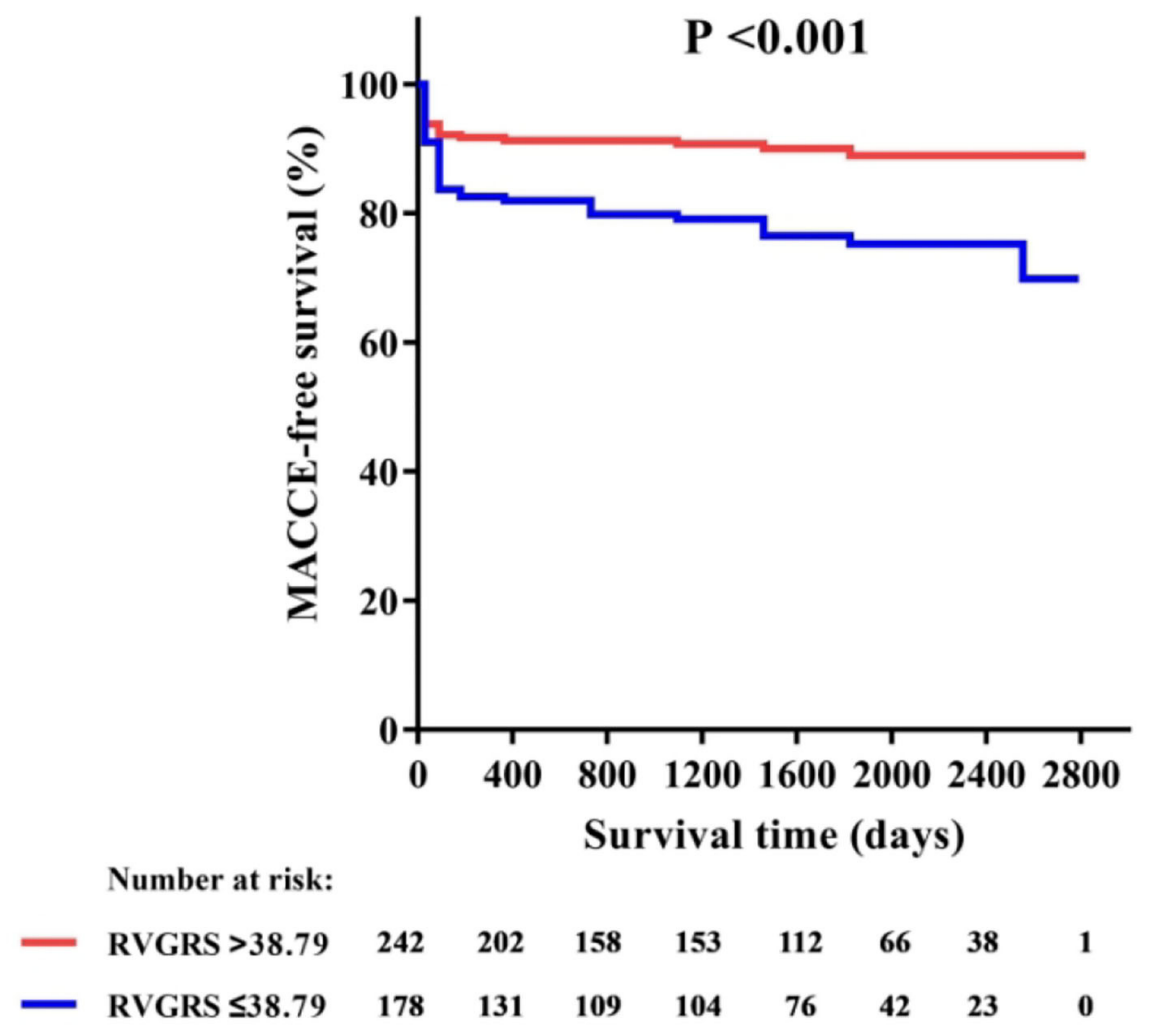
Kaplan-Meier curve of MACCE-free survival in patients with or without RV-GRS impairment. GRS, global radial strain; MACCE, major adverse cardiac and cerebrovascular events.

**Figure 5:**
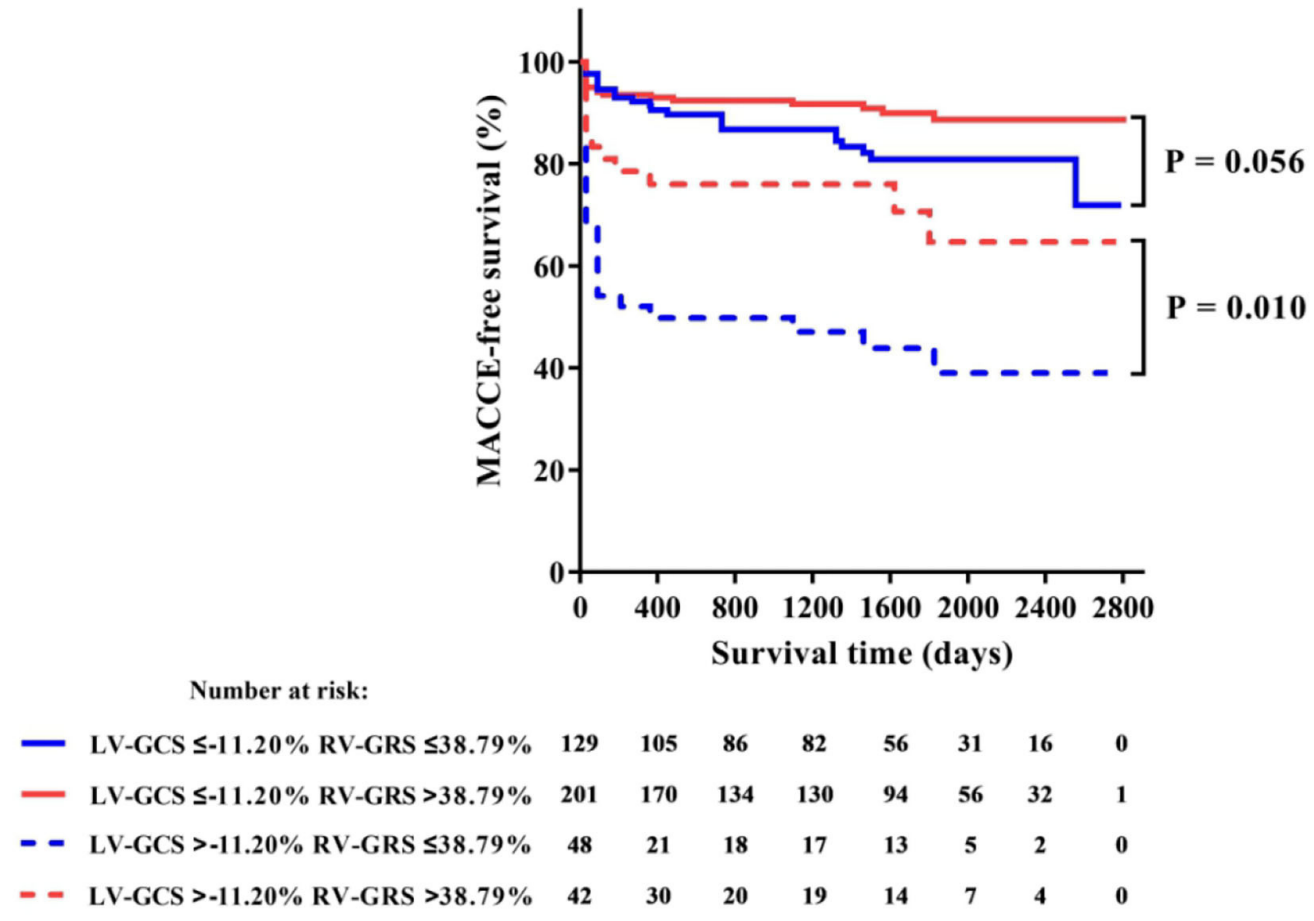
Kaplan-Meier curve of MACCE-free survival in patients with or without concomitant impairments of LV-GCS and RV-GRS. GCS, global circumferential strain; MACCE, major adverse cardiac and cerebrovascular events; GRS, global radial strain.

Among all RV strains, RV-GRS appeared to effectively predict MACCEs in this study (Youden index: 0.604; 95% CI: 0.533–0.674; *P* = 0.004), the optimal cut-off point was 38.79%. The MACCEs-free survival rate was significantly different between patients with RV-GRS beyond or below the cutoff (log rank *P* < 0.001) (Figure 4). Forty-eight patients had concomitantly reduction of RV-GRS and LV-GCS, these patients had a significantly worse prognosis than the 42 patients with reduced LV-GCS alone (*P* = 0.010). Patients with reduced RV-GRS but preserved LV-GCS also seemed to have a worse prognosis than patients with higher RV-GRS, but was not statistically significant (*P* = 0.056) (Figure 5). The addition of RV-GRS to LV-GCS improved the predictive power for MACCEs compared to LV-GCS alone (continuous NRI: 0.327; 95% CI: 0.095–0.558; *P* = 0.006).

### Factors That Influence RV-GRS

The incidence of reduced RV strain (<38.79%) in patients with occlusions in the left anterior descending branch (LAD), left circumflex branch (LCX), and right coronary artery (RCA) were 37.8, 37.5, and 51.5%, respectively. Tobacco use (67.4% of patients with decreased RV-GRS; *P* = 0.003), an occluded RCA (39.4% of patients with decreased RV-GRS; *P* = 0.002), and LV-GCS > −11.20% (27.1% of patients with decreased RV-GRS; *P* = 0.012) was significantly correlated with decreased RV-GRS (Table 4).

**Table 4:** Logistic regression analysis of RV-GRS impairment.

**Parameter**	**Univariate analysis**		**Multivariate analysis**	
	**HR (95% CI)**	***P***	**HR (95% CI)**	***P***
Age, y	1.027 (1.005, 1.050)	**0.016**		
Male, *n* (%)	0.407 (0.200, 0.828)	**0.013**		
Hypertension, *n* (%)	0.840 (0.568, 1.242)	0.383		
Diabetes, *n* (%)	1.344 (0.878, 2.056)	0.173		
Smoking, *n* (%)	0.547 (0.366, 0.818)	**0.003**	0.532 (0.353, 0.801)	**0.003**
Killip classification on admission, *n* (%)				
1	ref	–		
2	1.047 (0.655, 1.675)	0.847		
3	1.550 (0.648, 3.706)	0.324		
4	0 (0, –)	0.999		
TIMI flow post-PCI, *n* (%)				
0–2	0.504 (0.260, 0.974)	**0.042**		
3	ref	–		
Culprit vessels, *n* (%)				
LAD	Ref	–	Ref	–
LCX	0.992 (0.487, 2.023)	0.983	0.942 (0.453, 1.957)	0.873
RCA	0.569 (0.373, 0.870)	**0.009**	0.484 (0.309, 0.758)	**0.002**
Peak CK, U/L	1.000 (1.000, 1.000)	**0.041**		
Peak hs-cTnI, ng/ml	0.996 (0.990, 1.001)	0.141		
Peak BNP, pg/ml	1.000 (1.000, 1.001)	0.380		
LV-GRS, %	1.011 (0.985, 1.037)	0.424		
LV-GCS, %	0.953 (0.907, 1.002)	**0.059**	0.934 (0.886, 0.985)	**0.012**
LV-GLS, %	0.982 (0.924, 1.045)	0.569		
LV-EDV, ml	0.999 (0.993, 1.005)	0.782		
LV-ESV, ml	0.997 (0.990, 1.005)	0.492		
LV-EF, %	1.008 (0.992, 1.025)	0.309		
LV-IS, % LVMM	0.992 (0.976, 1.007)	0.288		
LV-MVO, % LVMM	0.935 (0.873, 1.001)	**0.055**		
LV IMH, *n* (%)	1.147 (0.776, 1.694)	0.491		

*BNP, B-type natriuretic peptide; CK, creatine kinase; EDV, end-diastolic volume; EF, ejection fraction; ESV, end-systolic volume; GCS, global circumferential strain; GLS, global longitudinal strain; GRS, global radial strain; hs-cTnI, high-sensitivity cardiac troponin I; IMH, Intramyocardial hemorrhage; LVMM, left ventricular myocardial mass; MVO, microvascular obstruction; PCI, percutaneous coronary intervention; TIMI, thrombolysis in myocardial infarction; IS, infarct size; SV, stroke volume. Factors with bold values are included in the multivariate analysis*.

### Reproducibility of CMR Measurements

We found a high consistency in both intra- and inter-observer variabilities of the CMR measurements. Detailed Intraclass correlation coefficient data and Bland-Altman graphics can be found in Supplementary Table 1 and Supplementary Figure 1.

## Discussion

This study focused on the relationship between CMR-derived myocardial strain data and STEMI prognosis. We found that post-STEMI LV strain is a key determinant of patient prognosis and that LV-GCS > −11.20% in the acute phase of STEMI is an independent predictor of long-term MACCEs. We also found that concomitantly reduction of RV-GRS and LV-GCS resulted in a worsened prognosis. Combinative assessment of RV-GRS to LV-GCS significantly improved the predictive power for MACCEs than LV-GCS alone. Besides, tobacco use, occlusions in the RCA, and impaired LV-GCS were associated with the decrease of RV strain.

Patients with STEMI have a highly diverse prognosis. Myocardial strain has emerged as a more useful marker than LVEF to reflect both global and regional myocardial function (3). Echocardiography is the most convenient method to measure myocardial strain. However, due to the high-quality images and computer-oriented imaging in comparison to echocardiography (5, 10), CMR-based strain measurements are more reliable and have become the reference method of strain assessment (16, 17).

Almost all studies focusing on myocardial strain have demonstrated that LV strain is a determining factor in the prognosis of patients with STEMI. In our study, LV-GLS, LV-GCS, and LV-GRS were significantly reduced in patients who experienced MACCEs compared to those who did not, and LV-GCS was identified as an independent predictor of MACCEs. This result is consistent with those of Nucifora's study (18). In other studies, CMR-derived LV-GLS had been reported to independently predict long-term prognosis (4, 5). The varied predictive powers of the three individual LV-strain indices among studies have not been clarified yet. However, LV-GCS is more accurate and reproducible than the other two indexes when compared with CMR tagging measurement (18–20). As demonstrated by Buss, the CMR-derived LV-GCS could distinguish an infarction area similar to that recognized by LGE (21). Neizel also found that LV-GCS could predict LVEF changes 6 months after the occurrence of STEMI (22). Both of them are well-recognized determinants for STEMI prognosis.

In contrast, the role of the RV function in the risk stratification of STEMI patients had only limited evidences that mostly provided by echocardiographic studies. In the current study, we found that lower RV strain was associated with increased MACCEs and improved the accuracy of risk stratification only based on the impairment of LV strain. Besides, our finding that reduction of RV strain could be found in all-location infarctions may be an important supplement to previous studies that had only researched RV strain in patients with RV or inferior infarctions (23, 24). The mechanism of RV strain impairments in non-inferior infarctions might be similar to right heart dysfunction due to left heart failure (25). Interestingly, we also found that tobacco use is associated with RV dysfunction. Since chronic obstructive pulmonary disease (COPD) in patients who smoke can cause pre-existing RV strain impairment (26), our results suggest that STEMI patients who smoke are more likely to have RV dysfunction, which do not conflict with the “smoker's paradox” theory claiming that smoking can lead to a more favorable left ventricular remodeling process (27).

The finding that concomitant reduction of RV and LV strain resulted in a worse prognosis than LV strain alone has important clinical implications. Although LV dysfunction is the most critical determinant for a patient's prognosis, our results suggest that a combinative assessment of RV function using CMR imaging analysis further increases the accuracy of risk stratification, especially in patients with decreased LV function.

## Limitations

This study has several limitations. First, the CMR images of study population were not consecutively included due to loss of follow-up, poor image quality, etc. Second, the follow-up CMR data of study population was limited, which hampered the dynamic monitoring of myocardial strain. Finally, this study didn't possess the images of quantitative T1 mapping as well as T2^*^ mapping which had been proposed for advanced infarct characterization with potential for improved risk stratification post-STEMI.

## Conclusions

In conclusion, concomitant impairment of both LV and RV strain is associated with a worse long-term prognosis than impaired LV strain alone. A combinative assessment of both RV and LV strains based on CMR images could improve risk stratification of patients with STEMI.

## Data Availability Statement

The original contributions presented in the study are included in the article/Supplementary Material, further inquiries can be directed to the corresponding author/s.

## Ethics Statement

The studies involving human participants were reviewed and approved by Shanghai Jiaotong University School of Medicine, Renji Hospital Ethics Committee. The patients/participants provided their written informed consent to participate in this study.

## Author Contributions

WL and HJ: analyzed images and data and drafted the manuscript. GH: concept development and critical review of the manuscript. DJ-X, ZJ-T, SB-Z, and YF-H: image analysis and critical review of the manuscript. AD-A-L, CB-H, XJ-R, and YY-N: collected images and critical review of the manuscript. DS, LZ, and WH-W: statistical analysis and critical review of the manuscript. PJ: all aspects of study. All authors read and approved the final manuscript and worked in the design or data collection of EARLY-MYO-CMR (NCT03768453) registry.

## Conflict of Interest

The authors declare that the research was conducted in the absence of any commercial or financial relationships that could be construed as a potential conflict of interest.
