# Supplementary material for: Impact of Concomitant Impairments of the Left and Right Ventricular Myocardial Strain on the Prognoses of Patients With ST-Elevation Myocardial Infarction
Source: Front Cardiovasc Med. 2021 May 31;8:659364. doi: 10.3389/fcvm.2021.659364 (PMC8200389; doi:10.3389/fcvm.2021.659364)
Supplement: Supplementary file 1 [file Data_Sheet_1.docx]

**Supplemental Table 1. Intraclass correlation coefficient of CMR strain analysis**

| CMR strain indexes | Intra-observer | Inter-observer |
| --- | --- | --- |
| LV-GRS, % | 0.973 (0.903, 0.993) | 0.931 (0.711, 0.983) |
| LV-GCS, % | 0.937 (0.761, 0.984) | 0.943 (0.790, 0.985) |
| LV-GLS, % | 0.965 (0.868, 0.991) | 0.918 (0.718, 0.979) |
| RV-GRS, % | 0.868 (0.572, 0.965) | 0.839 (0.485, 0.957) |
| RV-GCS, % | 0.944 (0.756, 0.987) | 0.837 (0.496, 0.956) |
| RV-GLS, % | 0.941 (0.788, 0.985) | 0.843 (0.509, 0.958) |

LV-GCS indicates left ventricular global circumferential strain; LV-GLS, left ventricular global longitudinal strain; LV-GRS, left ventricular global radial strain; RV-GCS, right ventricular global circumferential strain; RV-GLS, right ventricular global longitudinal strain; and RV-GRS, right ventricular global radial strain.


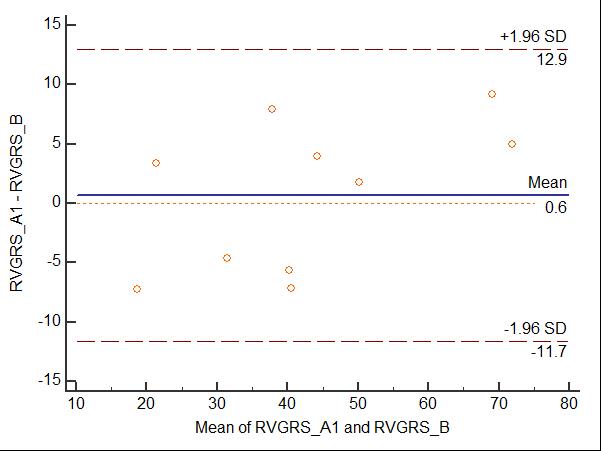

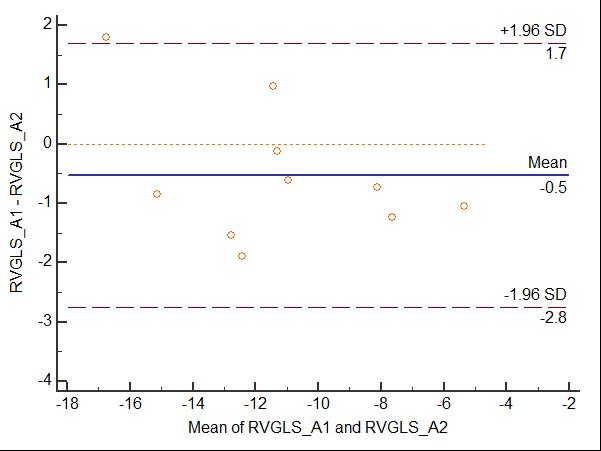

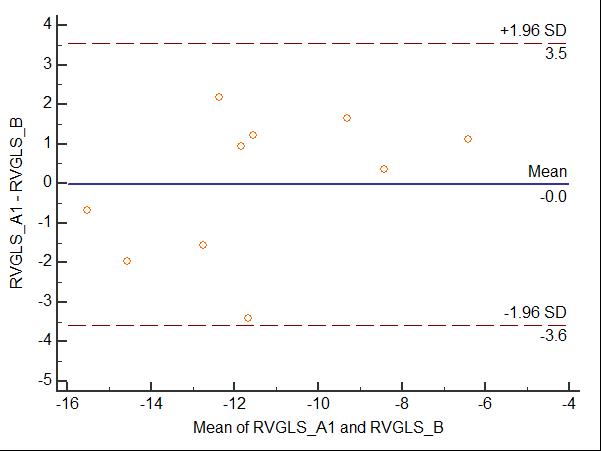

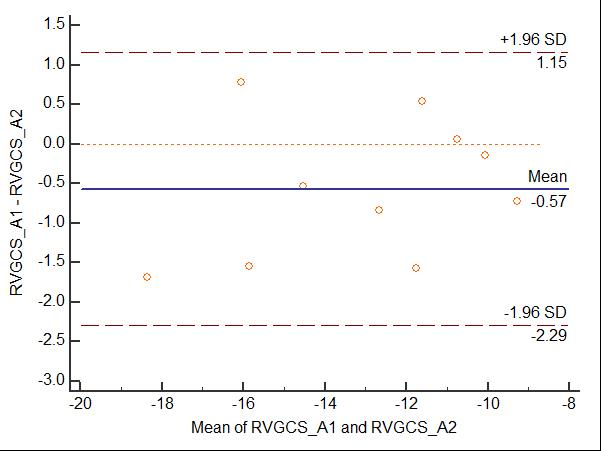

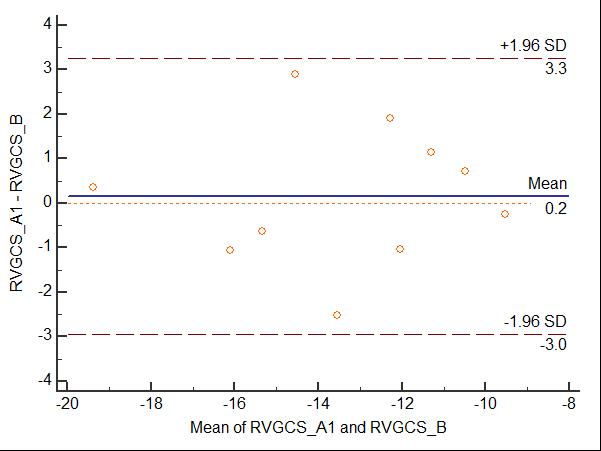

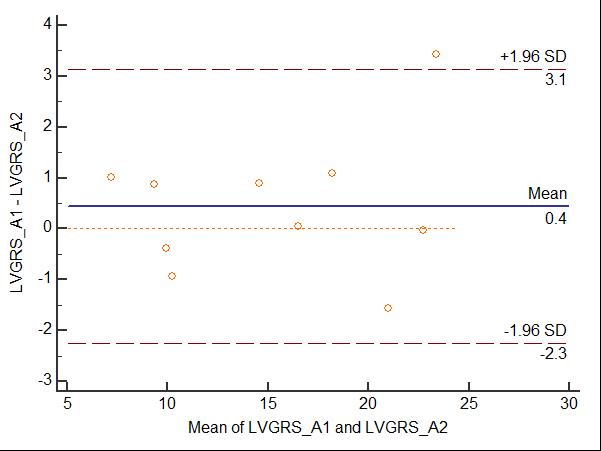

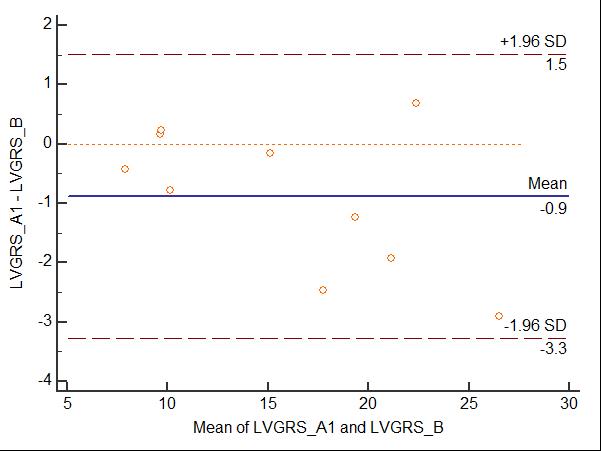

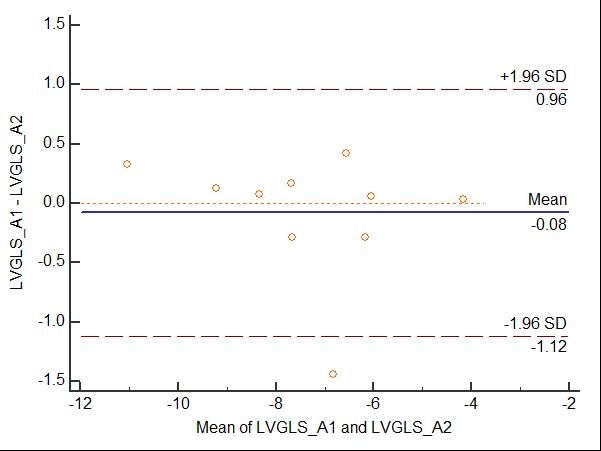

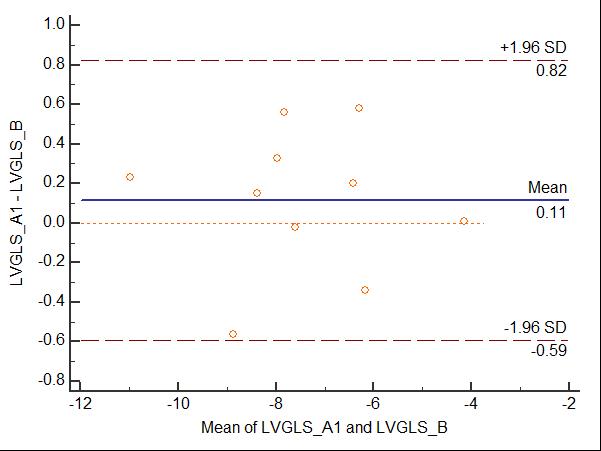

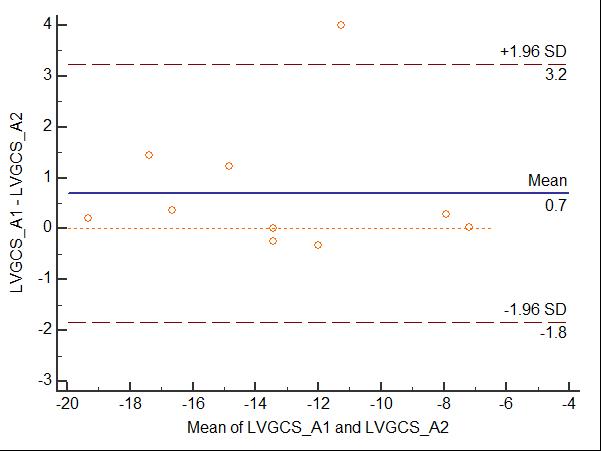

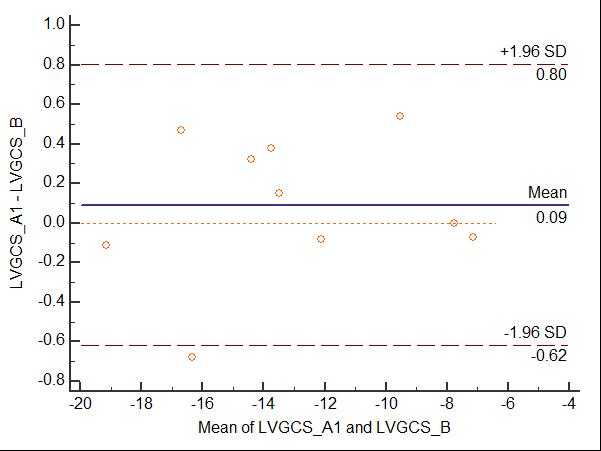

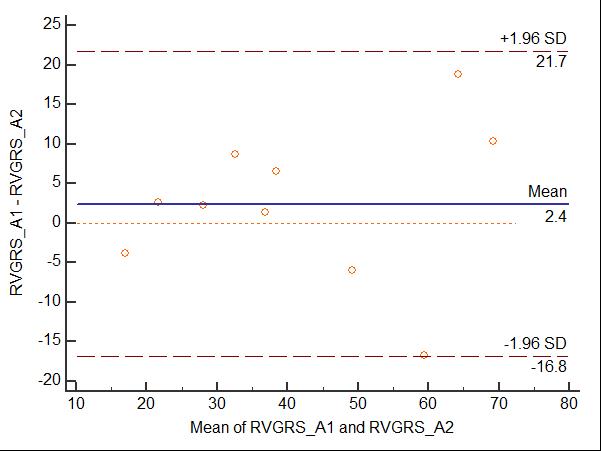


**Supplemental Figure 1. Bland-Altman analysis of LV and RV strain indexes.**
